# Effects of Dietary Vegetable Oils on Mammary Lipid-Related Genes in Holstein Dairy Cows

**DOI:** 10.3390/ani10010057

**Published:** 2019-12-27

**Authors:** Einar Vargas-Bello-Pérez, Carolina Geldsetzer-Mendoza, Nathaly Cancino-Padilla, María Sol Morales, Heidi Leskinen, Philip C. Garnsworthy, Juan J. Loor, Jaime Romero

**Affiliations:** 1 Departamento de Ciencias Animales, Facultad de Agronomía e Ingeniería Forestal, Pontificia Universidad Católica de Chile, Casilla 306, Santiago 6904411, Chile; clgeldsetzer@uc.cl (C.G.-M.); nlcancino@uc.cl (N.C.-P.); 2 Department of Veterinary and Animal Sciences, Faculty of Health and Medical Sciences, University of Copenhagen, Grønnegårdsvej 3, DK-1870 Frederiksberg C, Denmark; 3 Departamento de Fomento de la Producción Animal, Facultad de Ciencias Veterinarias y Pecuarias, Universidad de Chile, Av. Santa Rosa, La Pintana, Santiago 11735, Chile; smorales@uchile.cl; 4 Milk Production, Production Systems, Natural Resources Institute Finland (Luke), FI-31600 Jokioinen, Finland; heidi.leskinen@luke.fi; 5 School of Biosciences, The University of Nottingham, Sutton Bonington Campus, Loughborough LE12 5RD, UK; Phil.Garnsworthy@nottingham.ac.uk; 6 Mammalian NutriPhysioGenomics, Department of Animal Sciences and Division of Nutritional Sciences, University of Illinois, Urbana, IL 61801, USA; jloor@illinois.edu; 7 Laboratorio de Biotecnología en Alimentos, Unidad de Alimentos, Instituto de Nutrición y Tecnología de los Alimentos, Universidad de Chile, Avda. El Libano 5524, Macul, Santiago 7830490, Chile; jromero@inta.uchile.cl

**Keywords:** gene abundance, mammary gland, milk somatic cells, transcriptomic, olive oil

## Abstract

**Simple Summary:**

This study analyzed effects of vegetable oils fed to dairy cows on abundance of genes related to lipid metabolism in milk somatic cells (MSC). During 63 days (9 weeks), 15 cows were allocated to 3 treatments: a control diet with no added lipid and the same diet supplemented with olive oil (OO, 30 g/kg DM) or hydrogenated vegetable oil (HVO, 30 g/kg DM). Dietary oil supplementation (3% DM) had a modest nutrigenomic effect on biological functions such as acetate and FA activation and intra-cellular transport, lipid droplet formation, and transcription regulation in MSC. Results suggest that long-term dietary monounsaturated and saturated lipids could alter mRNA abundance in MSC from mid-lactating cows.

**Abstract:**

This study analyzed effects of vegetable oils fed to dairy cows on abundance of genes related to lipid metabolism in milk somatic cells (MSC). During 63 days, 15 cows were allocated to 3 treatments: a control diet with no added lipid the same diet supplemented with olive oil (OO, 30 g/kg DM) or hydrogenated vegetable oil (HVO, 30 g/kg DM). On days 21, 42 and 63, MSC were obtained from all cows. Relative abundance of genes involved in lipid metabolism in MSC from cows fed control on days 42 and 63 was compared with relative abundance at day 21 to evaluate fold-changes. Those genes without changes over the time were selected to analyze effects of OO and HVO. Compared with control, on day 42, *PLIN2* and *THRSP* were upregulated by OO. Compared with control, on day 21, HVO up regulated *ACACA*, down regulated *FABP3*, and on day 63 *THRSP* and *FABP4* were down regulated. Dietary oil supplementation (3% DM) had a modest nutrigenomic effect on different biological functions such as acetate and FA activation and intra-cellular transport, lipid droplet formation, and transcription regulation in MSC.

## 1. Introduction

Vegetable oils have been used in dairy diets to increase energy density of rations or to alter milk fatty acid (FA) profiles [1]. Dietary oil supplements affect mammary lipid metabolism partly through changes in lipogenic gene abundance. Piperova et al. [2] supplemented lactating cows with soybean oil and found that mammary activity of acetyl-CoA carboxylase (*ACACA*) and fatty acid synthase (*FASN*) both genes involved in FA synthesis and desaturation, decreased compared with a control diet (no fat supplementation). Jacobs et al. [3] evaluated the effects of feeding rapeseed oil, soybean oil, or linseed oil on stearoyl-CoA desaturase (*SCD1*, a gene involved in FA desaturation) abundance in the mammary gland of dairy cows and reported a significantly downregulation due to feeding unprotected soybean oil compared with rapeseed oil or linseed oil, and that was partially reflected by lower desaturase indices in milk. Angulo et al. [4] studied effects of polyunsaturated FA from plant oils (sunflower and linseed oils) and algae on mammary gene abundance and reported that milk fat profile was associated with down-regulation of both mammary lipogenic enzyme gene abundance (*SCD1* and *FASN*) and abundance of the regulatory element binding transcription factor (*SREBF1*; involved in the transcriptional regulation of lipogenesis). Vahmani et al. [5] studied the effects of pasture versus housing and marine oil supplementation on abundance of genes involved in lipid metabolism in mammary tissue of lactating cows and found that mammary mRNA abundance of *PPARG* and *FASN* were lower in grazing compared with cows in confinement. That was accompanied by reduced secretion of de novo synthesized FA in milk. More recently, Ibeagha-Awemu et al. [6] evaluated effects of supplementing mid-lactating cows with linseed oil and safflower oil (both unsaturated but with different FA profiles) on gene abundance and metabolic pathways. Compared with safflower oil, linseed oil had a greater impact on mammary gland transcriptome by affecting more genes, pathways, and processes. 

Mathews et al. [7] reported that compared to an unsupplemented lipid diet, prolonged (7 weeks) lipid supplementation with palmitic acid in mid-lactating dairy cows can maintain increases in milk fat yield but is unknown if that effect is due to changes/adaptations in gene abundance. Studies dealing with gene abundance in mammary tissue of cows fed added lipid typically last up to 10 weeks only [2,3,4,6], and mechanisms involved in a longer-term response are not considered. Therefore, it may be possible that changes in mRNA abundance of genes involved in lipid synthesis and secretion would be more clearly observed after relatively long periods of lipid supplementation. 

The molecular mechanisms underlying relatively long-term effects (9 weeks) in cows fed different vegetable oils are not well characterized. Total RNA extracted from milk epithelial cells and milk fat globules have been used to assess transcriptional activity of secretory mammary epithelium in livestock [8]. Due to animal welfare concerns among other issues such as risk of infections, instead of percutaneous mammary gland biopsy, alternative sampling approaches to study gene abundance at the mammary gland level have been proposed: milk somatic cells, laser microdissected mammary epithelial cells, milk fat globules and antibody-captured milk mammary epithelial cells [9]. Compared with biopsies, analysis of milk somatic cells (MSC) is an accessible method [10] especially when dynamic studies involving multiple sampling time points on the same animal are required [11]. Canovas et al. [9] reported that milk somatic cells are representative sources of RNA in mammary gland tissue, and their isolation is an effective and simple method to study the mammary gland transcriptome.

In general, nutrigenomics research using milk somatic cells (MSC) as an approach to evaluate candidate genes associated with lipid metabolism in mammary gland is scarce. For this reason, the aim of the current study was to determine effects of dietary vegetable oils on abundance of genes related to lipid metabolism in dairy cows using MSC. Degree of FA saturation in dietary lipids exert different effects on mammary gland gene abundance [6], thus, treatments were unrefined olive oil residues (OO; as a monounsaturated FA source) and hydrogenated vegetable oil (HVO; as a saturated FA source).

## 2. Materials and Methods 

### 2.1. Animals and Experimental Diets 

Animal care and procedures were carried out according to the guidelines of the Animal Care Committee of the Pontificia Universidad Católica de Chile. The study was conducted at the Estación Experimental Pirque of the Pontificia Universidad Católica de Chile (ID 150730013). Fifteen pregnant Holstein cows averaging 189 ± 28 days in milk at the beginning of the study were assigned to three treatment groups based on body condition score (BCS) and milk yield. Before commencing the study, average BCS for the 3 groups was 2.8 ± 0.3, 3.0 ± 0.0, and 2.8 ± 0.3. Milk yield for the 3 groups averaged 33.8 ± 3.5, 32.9 ± 3.5, and 32.5 ± 3.5 kg/d. Details of diets and management are presented in a companion paper [1]. Briefly, during 63 days all cows received a basal diet (65% forage and 35% concentrate) distributed as a total mixed ration (Table 1). The control or basal diet contained no added lipid (*n* = 5 cows); treatment diets were supplemented with OO (*n* = 5 cows; unrefined olive oil; 30 g/kg DM) or HVO (*n* = 5 cows; manufactured from palm oil; 30 g/kg DM). Oils were mixed manually into the daily ration for each cow. 

### 2.2. Plasma Samples and Fatty Acid Analysis

On days 21, 42 and 63, blood samples (50 mL/cow) were obtained at 10:00 h (2 h after feeding) via jugular puncture. Blood was transferred to tubes containing lithium heparin (BD Vacutainer; Franklin Lakes NJ, USA) and immediately centrifuged for 15 min at 3000× *g* (C-28A; BOECO, Hamburg, Germany) for harvesting plasma. Samples were stored at −80 °C until analyzed for FA profiles. Lipid extraction and methylation of plasma samples were done as reported previously [12]. A gas chromatograph (GC-2010) system (Shimadzu Scientific Instruments AOC-20 s, Columbia, MD, USA) equipped with a 100-m column (Rt-2560 column 100 m × 0.32 mm × 0.20 μm column; Restek, Bellefonte, PA, USA) was used. All GC conditions, FA methyl ester and reference standard have been reported previously [13].

### 2.3. Milk Somatic Cell Sampling

With the aim of maximizing the number of MSC present in milk, sample collection was performed four hours [8] after routine morning milking (08:00 h) on days 21, 42 and 63 (approximately 150 mL of milk per quarter from each cow). Udder cleaning was performed with special care: first, udders were cleaned with water and soap; then, they were disinfected with chlorhexidine-based soap; lastly, teats were cleaned with RNAseZap (Ambion, Austin, TX, USA). Sterile gauze was used to cover the collection container during milk sampling. Milk was transferred from the collecting container to RNAse-free 50 mL tubes after collection.

From each cow at each sampling period, 50 mL of milk was used for RNA extraction. The pellet of MSC was obtained as described by Wickramasinghe et al. [14] and Suárez-Vega et al. [11] with modifications. Briefly, the MSC was pelleted by centrifugation at 540× *g* in 50 mL RNAse free sterile tubes for 10 min at 4 °C in the presence of a final concentration of 0.5 mM of EDTA. After centrifugation, the supernatant was discarded. During this step, a fatty layer usually formed at the top of the tube, which was removed using a sterile pipette tip. The cell pellet was then washed in PBS (pH 7.2) with 0.5 mM EDTA and centrifuged at 540× *g* in 15 mL RNAse free sterile tubes for 10 min at 4 °C. The last step was repeated until the fatty layer was totally removed. Once the pellet was clean, it was re suspended in 500 μL of Trizol (Invitrogen, Carlsbad, CA, USA) and homogenized. RNA extraction continued following a standard Trizol protocol (Invitrogen, Carlsbad, CA, USA).

### 2.4. RNA Extraction

Relative abundance of genes involved in lipid metabolism in MSC from cows fed control on days 42 and 63 was compared with relative abundance of day 21 to evaluate fold-change. The RNA was extracted using QIAzol Lysis Reagent (Qiagen Inc., Valencia, CA, USA) according to the manufacturer’s protocols. RNA quality and quantity were assessed by 1% agarose gel electrophoresis (RIN ≥ 7) and RNA quantification was measured fluorometrically using the Qubit RNA HS Assay Kit in the Qubit Fluorometer 3.0 (Invitrogen Co., Carlsbad, CA, USA) (Figure S1). Samples were treated with RQ1 RNase-Free DNase (Cat. No. M6101; Promega®, Madison, USA) to avoid genomic DNA amplification, and the absence of genomic DNA was confirmed by polymerase chain reaction (PCR) on the treated RNA. 

The first-strand cDNA synthesis was run on a SureCycler 8800 Thermal Cycler (Agilent Technologies Inc., Santa Clara, CA, USA) and performed using the ImProm-II® Reverse Transcription System (Promega®, Madison, WI, USA). Total RNA was combined with 0.5 µg/reaction oligo (dT)15 primer (Cat. No. C1101; Promega®, Madison, WI, USA) to a final volume of 5 µL and was incubated at 70 °C for 5 min. Next, 15 µL of transcription mix (4.6 µL of ImProm-II 5 × Reaction Buffer, 2.25 mM of MgCl2, 0.5 mM each of dNTP and Recombinant RNasin® Ribonuclease Inhibitor (Promega®, Cat. No. N2511, Madison, WI, USA), in the amount of 0.5 µL, and 1 µL ImProm-II Reverse Transcriptase (Promega®, Cat. No. A3802, Madison, WI, USA) was added. Following addition of transcription mix, the reaction was maintained at 25 °C for 5 min and was then transferred to 42 °C for 60 min. Reverse transcription reactions were stopped by heating the mixture at 70 °C for 15 min. cDNA was stored at −80 °C until use.

### 2.5. Gene Abundance

Genes and primer-pairs used in the current study are listed in Table 2 and the quantitative PCR performance for all genes including internal controls is shown in Table S1. The target genes are related to the following biological functions: FA import into cells (*LPL*, *VLDLR*) FA synthesis and desaturation (*ACACA*, *FADS2*, *FASN*, *SCD1*), acetate and FA activation and intra-cellular transport (*ACSL1*, *ACSS2*, *FABP3*, *FABP4*, *FATP*), triacylglycerol synthesis (*DGAT1*, *DGAT2*, *LPIN1*), lipid droplet formation (*PLIN2*) regulation of transcription (*INSIG1*, *PPARG*, *SCAP*, *SREBF1*, *THRSP*). For the normalization of cDNA loading, all samples were run in parallel using the following housekeeping genes [15]: *GAPDH* (glyceraldehyde 3-phosphate dehydrogenase), *EIF3K* (eukaryotic translation initiation factor 3 subunit K) and *UXT* (ubiquitously expressed prefoldin like chaperone).

The relative mRNA abundance levels of the target genes and the housekeeping genes were quantified using real-time PCR analysis with AriaMx^®^ (Agilent Technologies, Santa Clara, CA, USA). The amplification of specific PCR products was performed using LightCycler 480 SYBR Green I Master^®^ (Cat. No 4887352001; Roche, Indianapolis, IN, USA) according to the manufacturer’s instructions. All cDNA samples were analyzed in triplicate. The amplification protocol was as follows: one initial step at 95 °C for 10 min (denaturation and enzyme activation) followed by 40 cycles at 95 °C for 15 s, 60 °C for 1 min. After amplification, a melting curve analysis was performed over a range of 65–95 °C to verify that a single PCR product was generated at the end of the assay. The PCR primer efficiency (E) was calculated for each gene fluorescence curve with LinRegPCR 12.18 software.

### 2.6. Statistical Analysis

A model including diet, time, and diet × time as fixed effects and cow within treatment as random effect was used to examine differences in plasma FA profiles. All data were analyzed using the MIXED procedure in SAS (SAS Institute Inc., Cary, NC, USA). Least squares means were separated using the PDIFF (Piecewise Differentiable) statement in SAS.

The relative abundance software tool (REST^©^) was used to analyze qPCR results. This software incorporates PCR efficiency correction and reference gene normalization. It integrates a statistical analysis randomization algorithm to calculate the statistical difference of variation between two groups and a bootstrapping technique, which provides 95% confidence interval for abundance ratios [16]. To test the effect of diet, data on relative gene abundance were based on comparing control vs. OO and control vs. HVO at each sampling time (21, 42, and 63 days). To test the long-term effects of lipid supplementation, data on relative gene abundance were based on comparisons between day 21 vs. day 42, and day 21 vs. day 63. Relative quantification of gene abundance and the statistical analysis were performed with the REST^©^ software. The REST^©^ software uses a P(H1) test for the statistical analysis that involves a robust random sample reallocation to assess for significance.

## 3. Results and Discussion

### 3.1. Animal Performance

Details on production parameters, milk composition and milk FA profile were previously reported [1]. Briefly, compared with control and HVO, OO increased milk yield and decrease milk fat yield. OO increased contents of C18:1 *trans*-10, C18:1 *trans*-11, C18:1 *cis*-9, C18:3 *cis*-9, *cis*-12, *cis*-15, total MUFA and total PUFA in milk.

### 3.2. Fatty Acid Profile in Plasma

The most-abundant FA in plasma were C16:0, C18:0, C18:1 *cis*-9 and C18:2 *cis*-9, 12 which agrees with our previous studies with oil supplementation in dairy cows [12,13]. The main significant effects on plasma were observed with OO (Table 3). Normally, when unprotected oils (such OO) high in unsaturated FA are fed to dairy cows, an increase in biohydrogenation intermediates [17] will be transported in the blood with its consequent secretion in the milk. 

In this study, compared with control and HVO, OO increased contents C15:1 *cis*-9, C18:1 *trans*-11, C18:1 *cis*-9, C18:3 *cis*-9, *cis*-12, *cis*-15, and total MUFA in plasma. Compared with control and OO, HVO increased C14:0 and had the lowest proportions for C18:1 *trans*-11. Furthermore, plasma contents of C18:1 *trans*-11, C18:2 *cis*-9, *cis*-12, C18:2 *cis*-9, *trans*-11 and total MUFA increased over time. These data are relevant because it shows FA transport dynamics in the blood which is one of the main factors controlling lipid utilization by tissues and, ultimately, milk FA profiles. 

Under the conditions of this study, we were able to detect only some C18:1 trans isomers (C18:1 *trans*-10 and C18:1 *trans*-11). C18:1 *trans*-10 has been reported to affect (anti-lipogenic effect) the abundance of genes involved in lipid metabolism at the mammary gland level [18]. This trans isomer was found in milk from OO, therefore, results in plasma FA partly explain the decrease in milk fat yield observed with OO reported previously [1].

### 3.3. Genes Selected to Study Fatty Acid Metabolism

Relative abundance of genes involved in lipid metabolism in MSC from cows fed control (no fat supplementation) on days 42 and 63 was measured using the relative abundance of day 21 as a reference condition (Table S2). Those genes (*ACACA*, *PLIN2*, *THRSP*, *FABP3* and *FABP4*) that did not change over time using the control diet, were selected to analyze the effects of control vs. OO and control vs. HVO in each sampling time (21, 42, and 63 days). This decision was made in order to analyze only those genes that were not up- or down-regulated and most likely not affected by days in lactation. Since our study lasted for 63 days, stage of lactation may be a factor affecting the abundance of the target genes [19,20]. 

### 3.4. Effects of OO and HVO on Lipid Metabolism-Related Genes in MSC

Relative abundance of genes involved in lipid metabolism in MSC from OO (Table 4) and HVO (Table 5) was performed using the control (no fat supplementation) as the reference condition in each sampling time (21, 42, and 63 days). The main biological processes in MSC that were affected by dietary oil supplementation were acetate and FA activation and intra-cellular transport, lipid droplet formation, and transcription regulation.

On day 21, *ACACA* was up regulated by HVO (*p* = 0.05) and the same tendency was observed in OO (*p* = 0.077). In non-ruminants, *ACACA* transcription is regulated by 4 promoters, and these in turn are regulated mainly by the glucose content in the cell, the insulin:glucagon ratio, and the levels of the T3 hormone in the blood [21,22]. Our results agree with the lack of differences in the abundance of *ACACA* at the mammary gland level reported by Piperova et al. [2] who fed dairy cows with soybean oil. Similarly, Bernard et al. [23] fed dairy goats with soybeans and did not observe changes in abundance of *ACACA* at both mammary gland and adipose tissue levels. Stable abundance of *ACACA* in this study, could partly explain, the lack of differences observed in the proportions of C4:0, C6:0, and C16:0 in milk reported in our companion paper [1]. Synthesis of short-chain FA and palmitic acid is mainly regulated by the action of this enzyme, which would be a critical point in the de novo synthesis [24,25].

With regard to HVO, changes occurred with genes that encode enzymes of the family of fatty acid binding proteins. Compared with control (no oil supplementation), HVO downregulated *FABP3* on day 21 (*p* = 0.007) and *FABP4* on day 63 (*p* = 0.047). FABP3 plays an important role in channeling palmitic and stearic acids for desaturation [24,25], as it provides stearoyl-CoA to *SCD* which then releases oleic acid to *FABP4* [26]. It has been suggested that a reduction in abundance of *FABP3* in cell culture (bovine mammary epithelial cells) generates a decrease in levels of *SREBP1* and *PPARG*, thereby affecting synthesis of milk fat. On the other hand, accumulation of fat droplets is reduced when FABP3 abundance is reduced. Although the details are not yet well known, it is believed that FABP3 could act directly by interacting with transcription factors or indirectly by signaling molecules [26].

According to Liang et al. [26], the addition of stearic and palmitic acids in mammary gland epithelial cell cultures generates an increase in *FABP3* abundance. In the present study the abundance of *FABP3* and *FABP4* may be explained by the relatively high contents of C16:0 (40 g/100g) and C18:0 (31 g/100g) of HVO. In the mammary gland, *FABP4* is primarily responsible for the variation in the proportion of medium chain FA and long chain FA content in milk [27]. Similarly, Nafikov et al. [28] reported that the abundance of *FABP4* and milk C10:0, C12:0 and C14:0 concentration were associated and that was explained by the role of *FABP4* in transporting FA hydrolyzed from adipose tissue triglyceride (TAG) for milk TAG biosynthesis within mammary epithelial cells.

Thyroid hormone-inducible hepatic protein (*THRSP*) was upregulated on days 21 and 42 with OO (Table 4), whereas this protein was upregulated on day 42 and downregulated on day 63 with HVO (Table 5). Cui et al. [29] reported that dairy cow mammary glands producing milk with high fat content could have high *THRSP* mRNA and protein levels. They also observed that mammary epithelial cells with overexpressed *THRSP* can increase TAG levels and enhanced abundance of *FASN*, *PPARG*, and *SREBP1*, resulting in a critical role in de novo lipogenesis and consequently influencing milk fat synthesis. In this study, however, no difference was observed in milk C10:0, C12:0, and C14:0 between control and HVO.

Abundance of *THRSP* is reduced when there is milk fat depression or when mammary gland epithelial cells are cultured with CLA *trans*-10, *cis*-12 [11,30]. However, in the present study, our results are contradictory since *THRSP* was up regulated during days 21 and 42. We reported [5] that OO led to higher milk yield and lower fat yield compared with control and HVO. A dilution effect rather than milk fat depression process may explain those results and this is partly supported by the gene abundance observed for *THRSP*. 

Lipid in milk arises from secretion of triglyceride-rich cytoplasmic lipid droplets (CLD) from mammary epithelial cells. *Plin2*/Adipophilin (*ADFP*) is a member of the perilipin family of CLD binding proteins [31]. *PLIN2* abundance in mammary tissue increased during lactation [19]. *PLIN*2 was upregulated on day 42 with OO. In this study, pregnant cows in mid-lactation were used, thus, it is possible that animals experienced an increase in mammary gland development, which is related to increases in abundance of *PLIN*2. In addition, *PLIN2* has an important role in regulating formation and secretion of cytoplasmic lipid droplets in mice [32]. Based on that, in this study, the reduction in milk fat yield observed in OO may be explained because fat droplets would be accumulating inside the cells and would not have been secreted in the same way as in the control and HVO treatments. It is also possible that OO had a lipogenic effect that was not maintained during 9 weeks, which may explain how this oil elicits a short-term impact on lipogenesis. An alternative approach to understand the effects of OO would be to study effects at the rumen level (i.e., microbiome), as there might be microbial adaptations to this dietary lipid.

### 3.5. Long-Term Effects in the Relative Abundance of Lipid-Related Genes in MSC

An important feature of this study is that we were able to detect some long-term effects on abundance of lipid-related genes in MSC from dairy cows fed with different dietary oils. If we consider all target genes, in general, relative abundance of lipid-related genes had a similar pattern between control and HVO over the 63 days of supplementation (Tables S2 and S3), which partly explains results for overall animal performance and milk FA profile. Those genes were related to the following functions: entry of FA to the cell (*VLDLR*—downregulated), synthesis and desaturation of FA (*FASN*—upregulated), activation and transport of FA (*ACSL1*—upregulated, *FABP3*—unchanged, *FABP4*—unchanged), triglyceride synthesis (*DGAT1* and *DGAT2*—both upregulated), and transcription regulation (*SREBF1*—upregulated and *THRSP*—unchanged).

In the control group, abundance of *FASN* increased at day 63 (cows were around 252 days postpartum), which contradicts Bionaz and Loor [19], who reported that on day 240 relative to parturition, this gene, along with *SCD* and *FABP3*, are regulated downwards. In the present study (Table S2), with unsupplemented cows, a decrease in SCD abundance was observed at day 42 and then remained stable. Abundance of *FABP3*, remained stable throughout the study. In agreement with our findings, Shi et al. [33] reported the same temporal pattern for abundance of *FABP3* and *SCD* in mammary tissue explants from goats.

The temporal downregulated *SREBF1* pattern observed on day 63 in the OO group (Table S4) normally occurs during supplementation with polyunsaturated FA sources. This leads to a decrease in activity of genes related to synthesis and desaturation of FA and FA import into cells (i.e., *FASN* and *LPL*), which are regulated by this transcription factor [20,34]. Similarly, in mice [35] fed with high amounts of unsaturated FA, hepatic abundance of *SREBF1* was suppressed together with the proportion of de novo FA in milk. 

Synthesis of milk fat at the mammary gland level requires coordination of multiple biochemical processes, and various transcription regulatory factors participate in the processes. Among those are *SREBP1* and *PPARG* (Table S4), which are pivotal for regulation of genes involved in metabolism of FA [36]. Overall, our results suggest that in long-term nutritional interventions unsaturated FA, such as OO, will suppress key transcription factors that play a pivotal role in biosynthesis of milk fat.

Some factors need to be taken into account when interpreting the relative abundance data of this study. We observed marginal changes in relative abundance of lipid-related genes in MSC, which could be related to the low number of animals used for each treatment. Animals received a modest amount of lipid supplement (3% DM), which was enough to induce changes in milk FA profile without compromising overall performance of cows. 

In general, relative abundance of *ACACA*, *PLIN2*, *THRSP*, *FABP3* and *FABP4* did not change significantly during the first 42 days of supplementation with OO and HVO. It seems that when animals are supplemented at 3% DM with dietary oils, changes will occur during the first 6 weeks of supplementation, and afterwards genes will revert to basal relative abundance. Similarly, in a companion paper [15], we reported that OO and HVO have a relatively mild effect on abundance of lipogenic genes in subcutaneous adipose tissue in mid-lactating cows.

Lastly, it is noteworthy mentioning that MSC are not only useful for gene expression studies but also, they could be used for studies related to udder health as they can explain inflammatory processes, cell death or apoptosis [9]. MSC are also suitable for studying lactogenesis, immunity transmission, cancer research and infection by viruses [8].

## 4. Conclusions

Dietary oil supplementation (3% DM) during 63 days (9 weeks) had a modest nutrigenomic effect on biological functions such as acetate and FA activation and intra-cellular transport, lipid droplet formation, and transcription regulation in MSC. Our results suggest that monounsaturated and saturated dietary lipids could lead to differential gene abundance in MSC. Our data also point at molecular adaptations when mid-lactating cows are supplemented long-term with oils.

## Figures and Tables

**Table 1:** Ingredients of control, olive oil (OO), and hydrogenated vegetable oil (HVO) dietary treatments.

Ingredient Composition (% DM)	Diet		
	Control	OO	HVO
Fresh alfalfa	28.9	28.9	28.9
Corn silage	27.0	27.0	27.0
Malt distillers	23.1	23.1	23.1
Corn grain	8.3	8.3	8.3
Wheat bran	6.2	6.2	6.2
Alfalfa hay	2.6	2.6	2.6
Soybean grain	2.0	2.0	2.0
Rapeseed meal	1.5	1.5	1.5
Vitamin and mineral premix ^a^	0.4	0.4	0.4
Olive oil	0	3.0	0
Hydrogenated vegetable oil	0	0	3.0

^a^ Contained per kg: 25 g of P; 80 g of Ca; 25 g of Mg; 1.6 g of S; 300,000 IU of vitamin A; 50,000 IU of vitamin D_3_ and 1600 IU of vitamin E.

**Table 2:** Gene symbol, name, sequence and size of the 20 genes measured in milk somatic cells.

Gene	Name	Sequence	Bp (Size)	Ref.
*ACACA*	Acetyl-CoA carboxylase alfa	F: 3709 CATCTTGTCCGAAACGTCGAT R: 3809 CCCTTCGAACATACACCTCCA	101	B
*ACSL1*	Acyl-CoA Synthetase Long Chain Family Member 1	F.1929 GTGGGCTCCTTTGAAGAACTGT R.2048 ATAGATGCCTTTGACCTGTTCAAAT	120	A
*ACSS2*	Acyl-CoA Synthetase Short Chain Family Member 2	F. 1871 GGCGAATGCCTCTACTGCTT R. 1970 GGCCAATCTTTTCTCTAATCTGCTT	100	B
*PLIN2 (ADFP)*	Adipose Differentiation-Related Protein	F. 161 TGGTCTCCTCGGCTTACATCA R. 241 TCATGCCCTTCTCTGCCATC	81	B
*DGAT1*	Diacylglycerol O-acyltransferase Homolog 1	F. 190 CCACTGGGACCTGAGGTGTC R. 290 GCATCACCACACACCAATTCA	101	B
*DGAT2*	Diacylglycerol O-acyltransferase Homolog 2	F: 389 CATGTACACATTCTGCACCGATT R: 488 TGACCTCCTGCCACCTTTCT	100	B
*FABP3*	Fatty Acid Binding Protein 3	F. 458 GAACTCGACTCCCAGCTTGAA R. 559 AAGCCTACCACAATCATCGAAG	102	A
*FABP4*	Fatty Acid Binding Protein 4	F: 401 TGGTGCTGGAATGTGTCATGA R: 501 TGGAGTTCGATGCAAACGTC	101	A
*FADS2*	Fatty acid desaturase 2	F. 642 AAAGGGTGCCTCTGCCAACT R. 742 ACACGTGCAGCATGTTCACA	101	B
*FASN*	Fatty acid synthase	F.6473 ACCTCGTGAAGGCTGTGACTCA R.6564 TGAGTCGAGGCCAAGGTCTGAA	92	B
*FATP (SLC27A6)*	Soluble Carrier Protein 27A	F: 861 GGCAAGGGCATGGATGATC R: 956 GCGGTAGTACCTGCTGTGCAC	96	A
*INSIG1*	Insulin Induced Gene 1	F. 523 AAAGTTAGCAGTCGCGTCGTC R. 630 TTGTGTGGCTCTCCAAGGTGA	108	C
*LPIN1*	Lipin 1	F. 147 TGGCCACCAGAATAAAGCATG R. 247 GCTGACGCTGGACAACAGG	101	A
*LPL*	Lipoprotein Lipase	F. 327 ACACAGCTGAGGACACTTGCC R. 427 GCCATGGATCACCACAAAGG	101	B
*PPARG*	Peroxisome Proliferator Activated Receptor Gamma	F. 135 CCAAATATCGGTGGGAGTCG R. 235 ACAGCGAAGGGCTCACTCTC	101	B
*SCAP*	SREBF Chaperone	F. 1188 CCATGTGCACTTCAAGGAGGA R. 1295 ATGTCGATCTTGCGTGTGGAG	108	C
*SCD*	Stearoyl-CoA desaturase	F. 809 TCCTGTTGTTGTGCTTCATCC R. 909 GGCATAACGGAATAAGGTGGC	101	A
*SREBF1*	Sterol Regulatory Element Binding Transcription Factor	F. 2824 TGTCCACAAAAGCAAATCGC R. 2990 TGTCGACCACCTCTGGCTTC	101	A
*THRSP*	Thyroid Hormone Responsive	F. 631 CTACCTTCCTCTGAGCACCAGTTC R. 781 ACACACTGACCAGGTGACAGACA	151	C
*VLDLR*	Very Low Density Lipoprotein Receptor	F. 98 GCCCAGAACAGTGCCATATGA R. 200 TTTTCACCATCACACCGCC	103	B

A = Bionaz and Loor, 2008a; B = Bionaz and Loor, 2008b; C = Harvatine and Bauman 2006.

**Table 3:** Plasma fatty acid composition from cows fed control, olive oil (OO), and hydrogenated vegetable oil (HVO) dietary treatments.

Fatty Acid (g/100g of Fatty Acid)	Diets ^1^			SEM	*p*-Value		
	Control	OO	HVO		Diet (D)	Time (T)	D × T
C14:0	0.31 ^b^	0.31 ^b^	0.92 ^a^	0.13	0.010	0.972	0.984
C15:0	0.32	0.34	0.63	0.19	0.210	0.623	0.706
C15:1 *cis*-9	0.21 ^b^	0.88 ^a^	0.27 ^b^	0.56	0.042	0.443	0.394
C16:0	14.75	13.98	16.53	0.57	0.145	0.701	0.497
C17:0	0.71	0.61	0.45	0.16	0.275	0.275	0.725
C17:1 *cis*-9	0.33	0.11	0.2	0.15	0.372	0.154	0.155
C18:0	35.44	35.6	34.16	1.17	0.068	0.101	0.356
C18:1 *trans*-11	0.20 ^b^	0.27 ^a^	0.11 ^c^	0.04	0.007	<0.001	<0.001
C18:1 *cis*-9	0.32 ^b^	1.03 ^a^	0.90 ^b^	0.06	0.040	0.714	0.553
C18:2 *cis*-9, *cis*-12	46.01	44.31	44.2	2.04	0.616	0.026	0.416
C18:3 *cis*-9, *cis*-12, *cis*-15	0.37 ^b^	1.41 ^a^	0.56 ^b^	0.78	0.046	0.175	0.542
C18:2 *cis*-9, *trans*-11	1.02	1.15	1.07	0.41	0.970	<0.001	0.469
Σ Saturated fatty acids	51.5	50.8	50.4	1.47	0.751	0.446	0.364
Σ Monounsaturated fatty acids	1.07 ^b^	2.29 ^a^	1.46 ^b^	0.66	0.018	0.027	0.057
Σ Polyunsaturated fatty acids	47.40	46.84	45.84	1.82	0.689	0.071	0.511

^a–c^ Means in the same row with different superscript letters are significantly different (Diet *p* < 0.05). ^1^ Control, no fat supplement; OO, supplemented with 30 g/kg DM olive oil; HVO, supplemented with 30 g/kg DM hydrogenated vegetable oil. SEM: standard error of the mean.

**Table 4:** Relative abundance of genes involved in lipid metabolism in milk somatic cells from cows fed olive oil (OO) using the control (no fat supplementation) as the reference condition (control vs. OO, in each sampling time).

Gene	Day	Abundance	Standard Error	*p* Value	Regulation
*ACACA*	21	0.334	0.041–2.591	0.077	
	42	20.079	0.244–711.375	0.077	
	63	0.307	0.038–2.074	0.076	
*PLIN2 (ADFP)*	21	2.485	0.433–7.416	0.059	
	42	8.079	1.666–77.219	<0.001	UP
	63	0.639	0.231–2.658	0.319	
*THRSP*	21	54.151	21.098–146.235	<0.001	UP
	42	91.637	26.148–402.981	<0.001	UP
	63	1.252	0.005–72.772	0.859	
*FABP3*	21	1.266	0.294–3.534	0.565	
	42	1.266	0.200–13.812	0.713	
	63	0.586	0.206–3.130	0.199	
*FABP4*	21	0.472	0.112–2.295	0.159	
	42	1.009	0.200–7.091	0.986	
	63	0.7	0.158–3.246	0.514	

Control, no fat supplement; OO, supplemented with 30 g/kg DM olive oil; HVO, supplemented with 30 g/kg DM hydrogenated vegetable oil.

**Table 5:** Relative abundance of genes involved in lipid metabolism in milk somatic cells from cows fed hydrogenated vegetable oil (HVO) using the control (no fat supplementation) as the reference condition (control vs. HVO, in each sampling time).

Gene	Day	Abundance	Standard Error	*p* Value	Regulation
*ACACA*	21	0.225	0.022–3.169	0.051	UP
	42	2.021	0.114–54.081	0.506	
	63	0.608	0.119–2.447	0.349	
*PLIN2 (ADFP)*	21	0.842	0.223–3.708	0.732	
	42	1.445	0.199–21.925	0.555	
	63	1.799	0.623–4.701	0.069	
*THRSP*	21	1.722	0.079–27.974	0.531	
	42	8.096	1.274–30.976	<0.001	UP
	63	0.055	0.000–1.343	0.018	DOWN
*FABP3*	21	0.349	0.089–1.170	0.007	DOWN
	42	0.579	0.170–2.806	0.233	
	63	0.468	0.143–2.135	0.076	
*FABP4*	21	1.076	0.166–5.086	0.891	
	42	4.089	0.438–19.307	0.053	UP
	63	0.459	0.185–1.455	0.047	DOWN

Control, no fat supplement; OO, supplemented with 30 g/kg DM olive oil; HVO, supplemented with 30 g/kg DM hydrogenated vegetable oil.

## Supplementary Materials

The following are available online at https://www.mdpi.com/2076-2615/10/1/57/s1, Table S1: qPCR performance of target genes and internal controls (*GAPDH*, *EIF3K* and *UXT*), Table S2: Relative expression of genes involved in lipid metabolism in milk somatic cells from cows fed control (no fat supplementation) on days 42 and 63 using the relative abundance of day 21 as a reference condition, Table S3: Relative abundance of genes involved in lipid metabolism in milk somatic cells from cows fed with olive oil (OO) on days 42 and 63 using the relative abundance of day 21 as a reference condition, Table S4: Relative abundance of genes involved in lipid metabolism in milk somatic cells from cows fed with hydrogenated vegetable oil (HVO) on days 42 and 63 using the relative abundance of day 21 as a reference condition.
