# Supplementary material for: Effects of Dietary Vegetable Oils on Mammary Lipid-Related Genes in Holstein Dairy Cows
Source: Animals (Basel). 2019 Dec 27;10(1):57. doi: 10.3390/ani10010057 (PMC7023335; doi:10.3390/ani10010057)
Supplement: Supplementary file 1 [file animals-10-00057-s001.pdf]

Table S1. qPCR performance of target genes and internal controls (*GAPDH*, *EIF3K* and *UXT*)

Gene	Median Ct[1]	Median ΔCt[2]	Slope	(R^2)[3]	Efficiency[4]
ACACA	27.35	3.50	-3.32	0.997	1.99
ACSL1	28.83	4.98	-3.37	0.997	1.97
ACSS2	22.75	-1.10	-3.1	0.937	2.00
DGAT1	23.09	-0.77	-3.51	0.996	1.92
DGAT2	21.78	-2.07	-3.47	0.995	1.94
FABP3	29.05	5.20	-3.38	0.994	1.97
FABP4	29.22	5.36	-3.29	0.961	2.00
FADS2	27.00	3.14	-3.51	0.999	1.92
FASN	28.91	5.06	-3.76	0.998	1.84
INSIG1	30.14	6.28	-3.32	0.987	1.99
LPIN1	28.68	4.82	-3.53	0.995	1.91
LPL	27.09	3.23	-3.38	0.995	1.97
PLIN2 (ADFP)	27.24	3.39	-3.56	0.995	1.90
PPARG	22.55	-1.31	-3.43	0.996	1.95
SCAP	29.46	5.60	-3.54	0.998	1.91
SCD	28.27	4.41	-3.62	0.997	1.88
SLC27A6	25.12	1.26	-3.42	0.996	1.96
SREBF1	22.96	-0.89	-3.32	0.996	1.99
THRSP	20.06	-3.80	-3.44	0.995	1.95
VLDLR	22.36	-1.50	-3.38	0.995	1.97
GAPDH	21.09	-	-3.63	0.998	1.88
EIF3K	24.12	-	-3.4	0.994	1.96
UXT	26.36	-	-3.4	0.995	1.96

[1]The median is calculated considering all samples.

[2]The median of ΔCt is calculated as [Ct gene – geometric mean of Ct internal controls] for each sample.

[3]R^2 represents the coefficient of determination of the standard curve.

[4]Efficiency was calculated as [$10^{(-1\ /\ \mathrm{Slope})}$].

Table S2. Relative expression of genes involved in lipid metabolism in milk somatic cells from cows fed control (no fat supplementation) on days 42 and 63 using the relative abundance of day 21 as a reference condition

Gene	Day	Relative abundance	Standard error	P-value	Regulation
ACACA	42	0.329	0.020 - 4.69	0.274	
	63	2.485	0.396 - 11.4	0.101	
FADS2	42	0.167	0.072 - 0.389	<0.001	DOWN
	63	3.916	0.180 - 137.8	0.189	
FASN	42	1.392	0.058 - 44.7	0.772	
	63	11.268	0.888 - 155.4	<0.001	UP
SCD	42	0.171	0.024 - 1.96	<0.001	DOWN
	63	2.152	0.199 - 24.5	0.252	
ADFP (PLIN2)	42	0.716	0.141 - 5.91	0.642	
	63	2.251	0.307 - 10.7	0.117	
INSIG1	42	2.361	0.421 - 7.38	0.084	
	63	15.834	2.18 - 546.3	<0.001	UP
SCAP	42	0.046	0.016 - 0.109	<0.001	DOWN
	63	0.469	0.114 - 2.67	0.128	
SREBF1	42	1.815	0.152 - 12.8	0.363	
	63	10.979	1.79 - 108.3	<0.001	UP
THRSP	42	0.541	0.219 - 1.32	0.054	
	63	8.051	0.417 - 1.00	0.158	
PPARG	42	0.17	0.026 - 0.902	<0.001	DOWN
	63	2.744	1.09 - 7.92	<0.001	UP
DGAT1	42	0.562	0.037 - 12.3	0.583	
	63	9.123	1.07 - 62.9	<0.001	UP
DGAT2	42	1.917	0.266 - 8.45	0.247	
	63	8.284	0.631 - 75.0	<0.001	UP
LPIN1	42	2.313	0.098 - 54.7	0.432	
	63	19.127	2.72 - 117.0	<0.001	UP
LPL	42	5.565	0.924 - 33.1	<0.001	UP
	63	5.048	0.789 - 17.6	<0.001	UP
FATP	42	1.946	0.213 - 14.5	0.340	
	63	7.856	1.32 - 40.5	<0.001	UP
VLDLR	42	2.194	0.779 - 7.78	<0.001	DOWN
	63	4.497	1.26 - 14.8	<0.001	UP
ACSL1	42	9.677	2.61 - 30.2	<0.001	UP
	63	2.914	1.03 - 14.2	<0.001	UP
ACSS2	42	8.539	3.36 - 28.8	<0.001	UP
	63	2.46	0.620 - 9.34	<0.001	DOWN
FABP3	42	1.512	0.605 - 4.43	0.185	
	63	1.057	0.517 - 2.06	0.766	
FABP4	42	0.552	0.085 - 4.59	0.358	
	63	1.487	0.276 - 4.81	0.431	

Table S3. Relative abundance of genes involved in lipid metabolism in milk somatic cells from cows fed with olive oil (OO) on days 42 and 63 using the relative abundance of day 21 as a reference condition

Gene	Day	Abundance	Standard Error	P-Value	Regulation
ACACA	42	19.803	0.478 - 4.912.875	0.037	UP
	63	2.286	0.356 - 18.930	0.239	
FADS2	42	1.496	0.194 - 16.142	0.544	
	63	0.824	0.060 - 38.501	0.843	
FASN	42	1.994	0.168 - 74.288	0.445	
	63	0.041	0.002 - 0.515	0.001	DOWN
SCD	42	0.881	0.367 - 2.172	0.652	
	63	2.337	0.369 - 21.641	0.204	
ADFP (PLIN2)	42	2.326	1.296 - 4.679	0.001	UP
	63	0.579	0.168 - 1.900	0.16	
INSIG1	42	1.148	0.072 - 17.507	0.88	
	63	0.959	0.039 - 25.668	0.962	
SCAP	42	1.13	0.164 - 6.585	0.844	
	63	0.322	0.028 - 2.949	0.155	
SREBF1	42	0.593	0.098 - 9.093	0.489	
	63	0.136	0.025 - 0.992	0.003	DOWN
THRSP	42	0.915	0.245 - 3.531	0.875	
	63	0.186	0.043 - 1.169	0.015	DOWN
PPARG	42	1.409	0.149 - 8.976	0.615	
	63	0.35	0.048 - 2.145	0.098	
DGAT1	42	7.379	2.437 - 23.592	<0.001	UP
	63	0.168	0.022 - 1.072	0.01	DOWN
DGAT2	42	1.455	0.306 - 14.246	0.54	
	63	0.147	0.017 - 0.806	0.003	DOWN
LPIN1	42	2.442	0.469 - 14.763	0.117	
	63	0.537	0.122 - 2.350	0.233	
LPL	42	2.429	0.883 - 7.908	0.011	UP
	63	0.458	0.168 - 1.516	0.048	DOWN
FATP	42	2.455	0.569 - 9.738	0.068	
	63	1.187	0.559 - 3.334	0.569	
VLDLR	42	0.246	0.063 - 1.089	0.008	DOWN
	63	0.528	0.167 - 1.514	0.09	
ACSL1	42	28.495	4.217 - 180.201	<0.001	UP
	63	0.705	0.140 - 2.961	0.54	
ACSS2	42	2.636	0.734 - 16.940	0.1	
	63	0.268	0.044 - 2.152	0.036	DOWN
FABP3	42	1.512	0.260 - 5.239	0.519	
	63	0.489	0.089 - 1.677	0.197	
FABP4	42	1.181	0.390 - 3.941	0.672	
	63	2.206	0.293 - 13.372	0.175	

Table S4. Relative abundance of genes involved in lipid metabolism in milk somatic cells from cows fed with hydrogenated vegetable oil (HVO) on days 42 and 63 using the relative abundance of day 21 as a reference condition

Gene	Day	Abundance	Standard error	P- Value	Regulation
ACACA	42	2.955	0.361 - 26.228	0.135	
	63	6.719	1.797 - 47.276	0.023	UP
FADS2	42	0.256	0.043 - 1.896	0.044	DOWN
	63	0.172	0.015 - 3.036	0.071	
FASN	42	3.652	0.233 - 58.763	0.118	
	63	43.966	2.382 - 1.384.567	<0.001	UP
SCD	42	0.67	0.320 - 1.853	0.253	
	63	3.091	0.569 - 14.437	0.062	
ADFP (PLIN2)	42	1.228	0.282 - 5.536	0.607	
	63	4.811	1.849 - 12.244	<0.001	UP
INSIG1	42	1.103	0.196 - 3.505	0.83	
	63	0.935	0.159 - 2.972	0.876	
SCAP	42	0.305	0.057 - 2.118	0.055	
	63	1.473	0.129 - 17.374	0.569	
SREBF1	42	0.371	0.091 - 2.319	0.123	
	63	7.777	0.614 - 140.607	0.017	UP
THRSP	42	2.543	0.225 - 131.930	0.365	
	63	0.259	0.012 - 4.184	0.158	
PPARG	42	0.076	0.017 - 0.288	<0.001	DOWN
	63	1.206	0.167 - 7.902	0.755	
DGAT1	42	2.821	0.303 - 13.662	0.106	
	63	13.996	1.617 - 55.742	<0.001	UP
DGAT2	42	1.117	0.190 - 12.477	0.882	
	63	20.255	3.898 - 69.142	<0.001	UP
LPIN1	42	0.216	0.086 - 0.463	0.001	DOWN
	63	2.338	0.837 - 7.286	0.028	UP
LPL	42	1.118	0.519 - 2.527	0.718	
	63	3.974	1.917 - 12.158	<0.001	UP
FATP	42	3.259	1.358 - 7.567	0	UP
	63	3.183	1.029 - 14.975	0.018	UP
VLDLR	42	0.804	0.474 - 1.347	0.244	
	63	1.872	1.029 - 3.219	0.011	UP
ACSL1	42	11.963	1.774 - 223.026	0	UP
	63	5.809	0.947 - 108.280	0.023	UP
ACSS2	42	1.901	0.904 - 4.131	0.035	UP
	63	3.824	0.622 - 11.613	0.011	UP
FABP3	42	2.512	0.656 - 8.561	0.075	
	63	1.42	0.247 - 7.227	0.513	
FABP4	42	2.099	0.317 - 21.543	0.242	
	63	0.635	0.245 - 2.848	0.331	
